# Composition and diversity of gut microbiota in *Pomacea canaliculata* in sexes and between developmental stages

**DOI:** 10.1186/s12866-021-02259-2

**Published:** 2021-07-02

**Authors:** Lian Chen, Shuxian Li, Qi Xiao, Ying Lin, Xuexia Li, Yanfu Qu, Guogan Wu, Hong Li

**Affiliations:** 1 College of Biology and the Environment, Nanjing Forestry University, 210037 Nanjing, China; 2 Jiangsu Key Laboratory for Biodiversity and Biotechnology, College of Life Sciences, Nanjing Normal University, 210023 Nanjing, China; 3 Shanghai Academy of Agricultural Sciences, 201106 Shanghai, China

**Keywords:** *Pomacea canaliculata*, Gut microbiota, 16S rRNA gene, High-throughput sequencing

## Abstract

**Background:**

The apple snail, *Pomacea canaliculata*, is one of the world’s 100 worst invasive alien species and vector of some pathogens relevant to human health.

**Methods:**

On account of the importance of gut microbiota to the host animals, we compared the communities of the intestinal microbiota from *P. canaliculata* collected at different developmental stages (juvenile and adult) and different sexes by using high-throughput sequencing.

**Results:**

The core bacteria phyla of *P. canaliculata* gut microbiota included Tenericutes (at an average relative abundance of 45.7 %), Firmicutes (27.85 %), Proteobacteria (11.86 %), Actinobacteria (4.45 %), and Cyanobacteria (3.61 %). The female group possessed the highest richness values, whereas the male group possessed the lowest bacterial richness and diversity compared with the female and juvenile group. Both the developmental stages and sexes had important effects on the composition of the intestinal microbiota of *P. canaliculata.* By LEfSe analysis, microbes from the phyla Proteobacteria and Actinobacteria were enriched in the female group, phylum Bacteroidetes was enriched in the male group, family Mycoplasmataceae and genus *Leuconostoc* were enriched in the juvenile group. PICRUSt analysis predicted twenty-four metabolic functions in all samples, including general function prediction, amino acid transport and metabolism, transcription, replication, recombination and repair, carbohydrate transport and metabolism, etc.

**Conclusions:**

This study provided a general understanding of the diversity characteristics of intestinal microbial communities of *P. canaliculata*, and indicated that developmental stage and gender could both influence the intestinal microbes of *P. canaliculata.* Further study may focus on the interaction between the gut microbiota and their host.

**Supplementary Information:**

The online version contains supplementary material available at 10.1186/s12866-021-02259-2.

## Introduction

*Pomacea* is freshwater gastropods native to South America [1]. Several *Pomacea* species such as *Pomacea canaliculata*, *P. maculata* have been introduced and become severe invasive pests in many parts of the world including Asian countries, North America, islands of the Pacific, and Europe [2, 3]. *P. canaliculata* is listed as “100 world’ s worst invasive alien species” by the International Union for Conservative of Nature and the Invasive Species Specialist Group [4]. Due to its high fecundity (a female snail in its lifetime could averagely spawn 13,764 eggs and reproduce 6070 young snails [5]), fast growth and voracious appetite for vegetation, *P. canaliculata* is a notorious pest, causing serious damages to aquatic crops such as rice and lotus, as well as to wetland floral diversity and ecosystem functioning [6]. *P. canaliculata* is also a vector of a parasitic nematode *Angiostrongylus cantonensis*, which causes eosinophilic meningitis in humans [7]. *P. canaliculata* was introduced to China for commercial purposes in the early 1980 s, and widely distributed in most areas of southern part of China at present [7].

Recent studies have highlighted the ability of the gut bacteria of animals in multiple physiological processes of their hosts, primarily including digestion, nutrition, development, reproduction, immunity, and environmental resistance [8, 9]. Bacteria in the intestine of fish can produce various enzymes, such as protease, amylase, and lipase, for digestion [10]. Furthermore, the intestinal microorganisms of snail giant African snail (*Achatina fulica*) play an important role in cellulose decomposition [11]. It is increasingly recognized on the importance of intestinal microbial structure and function, especially the potential contribution to nutrition utilization of host [12].

Gut microbiomes can be influenced by host development and growth stages in some animals, such as zebrafish (*Danio rerio*), bovine and Atlantic salmon (*Salmo salar*) [13–15]. Moreover, gender has a significant correlation with the intestinal microbiota. The alpha diversity of the gut microbiome in three-spined stickleback (*Gasterosteus aculeatus*) and European perch (*Perca fluviatilis*) is different between sex [16]. The significant separation is found in the microbial communities between male and female fathead minnow (*Pimephales promelas*) based on beta diversity metrics [17].

Only limited studies have focused on the gut microbiota of *P. canaliculata*. Cheng et al. [18] investigated intestinal bacterial communities in *P. canaliculata* by denaturing gradient gel electrophoresis (DGGE). The results showed that the microflora of the male and female *P. canaliculata* gastric and intestinal contents was the same, containing 22 species of bacteria in all of the samples. Li et al. [19] compared the gut microbes of *P. canaliculata* at different gut sections via high-throughput sequencing of the 16 S rRNA gene. A total of 29 phyla and 111 genera of bacteria were identified in all of the samples. High-throughput sequencing opens up the possibility of conducting large-scale studies analyzing thousands of samples simultaneously to survey microbial communities at an unprecedented spatial and temporal resolution [20].

The differences related to developmental stages and gender in the gut microbiota of *P. canaliculata* have not been fully investigated. Diverse factors, such as diet, age, antibiotics, stress, psychological factors, mode of delivery, environmental factor, and exercise, can influence the status of the gut microbiota [21]. The influence of sex on the gut microbiota is not so clear when compared with other factors such as diet and medication [22]. Nevertheless, the effect of different genders on the gut microbiota and their interactions with other factors should be routinely analyzed. To better understand the bacterial community in the gut of this invasive snail and provide insight into their adaptive strategies in the environments, we investigated the differences related to developmental stages and gender in the gut microbiota of *P. canaliculata* by high-throughput sequencing in this study. Our study provided a framework for characterizing age-bacterial and sex-bacterial communities in *P. canaliculata* and supplemented information on intestinal content microbiota in *P. canaliculata*.

## Materials and methods

### Sample collection

*P. canaliculata* individuals were collected from an artificial pond located in Lishui City (E 119º54’, N 28º28’), Zhejiang province, China, in August 2019, including 11 adults (six males and five females, shell length ranged from 48.74mm to 57.76mm) and 15 juvenile snails (shell length ranged from 17.51mm to 19.71mm). All the testing snails sampled from artificial pond were preliminarily discerned by shell morphological analysis and using primers LCO1490/HCO2198 to amplify cytochrome c oxidase subunit I (COI) gene to identify *P. canaliculata* which could be used for the experiments [23, 24]. PCR amplification products were sequenced by Shanghai Sangon Biological Engineering Technology & Services Co., Ltd. *P. canaliculata* samples were divided into adult (≥ 30 mm shell length) and juvenile groups (10–25 mm shell length) [25]. In addition, the adult *P. canaliculata* were divided into male and female groups. The shell was removed from each snail after being wiped by 75 % ethanol three times and rinsing it twice in distilled water. Dissection was performed on ice using sterilized tools. Because the coiled gut of juvenile individuals was too small, samples of three snails were collected for sequencing [26]. Coiled gut content from three juvenile individuals were dissected and pooled to compose one juvenile biological sample and one adult individual were dissected and pooled to compose one adult biological sample [27]. Coiled gut contents were extracted carefully to avoid rupturing the gut wall. There were five and eleven samples representing the juvenile and the adult group for intestine microbial analysis respectively. The intestinal content of each sample was placed in sterile tubes and stored at -80 °C until DNA extraction.

### DNA extraction and high-throughput 16S rRNA gene sequencing

Extraction of microbial DNA from approximately 0.02 g of coiled gut contents extracted from each *P. canaliculata* sample was performed using the FastDNA® Kit (MP Biomedicals, CA, USA). The V3-V4 hyper-variable regions of the 16S rRNA genes were amplified by using the specific primer 338F (5’-ACTCCTACGGGAGGCAGCAG-3’) and 806R (5’-GGACTACHVGGGTWTCTAAT-3’). The PCR procedure was as follows: denaturation at 95 °C for 3 min, 27 cycles of 95 °C denaturation for 30 s, 55 °C annealing for 30 s, and 72 °C extension for 45 s, and a final extension at 72 °C for 10 min. PCR products were detected by 2 % agarose gel and further purified using the AxyPrep DNA Gel Extraction Kit (Axygen Biosciences, Union City, CA, USA) and quantified using QuantiFluor™-ST (Promega, USA). Purified amplicons were pooled and sequenced according to the standard protocols using the Illumina MiSeq platform (Majorbio Bio-Pharm Technology Co. Ltd., Shanghai, China).

### Statistical and bioinformatics analyses

The raw sequences were processed by Quantitative Insights into Microbial Ecology (QIIME) software (Version1.9.1, http://qiime.org/install/index.html). Raw fastq files were quality-filtered by Trimmomatic and merged by FLASH according to the following criteria: (i) The reads were truncated at any site receiving an average quality score < 20 over a 50 bp sliding window. (ii) Sequences overlapped longer than 10 bp were merged according to their overlap with mismatch no more than 2 bp. (iii) Sequences of each sample were separated according to barcodes (exactly matching) and primers (allowing 2 nucleotide mismatching), and reads containing ambiguous bases were removed. All of the remaining high-quality sequences were clustered into operational taxonomic units (OTUs) at a 97 % identity threshold using USEARCH (Version 7.0, http://drive5.com/uparse/) [28]. Rarefaction curves were generated based on normalized OTU numbers using Mothur software (Version 1.30.2, https://www.mothur.org/wiki/Download_mothur) [29]. Using RDP Native Bayesian Classifier (Version 2.11), the taxonomy of each 16S rRNA gene sequence was analyzed against the Silva 16S rRNA database (Version 132, http://www.arb-silva.de) [30]. Subsequently, BLAST search was further carried out on NCBI nucleotide collection (nr/nt) using the BLASTn algorithm to obtain information on taxonomic identity of the noranked OTUs on genus level, and the closest matches to bacterial strains were obtained.

Community diversity was estimated using alpha diversity indices including the Chao and Shannon indices using Mothur (Version 1.30.2). To display any discrepancy among the three groups (female; male; juvenile), nonmetric multidimensional scaling (NMDS) analysis was used based on the unweighted and weighted Unifrac distances and analysis of similarity (ANOSIM) based on 999 permutations. Based on Bray-Curtis distance, analysis of molecular variance (AMOVA), was performed to analyze the differences between the female and male group, and between the juvenile and adult group by Mothur software (Version 1.30.2). The differences in intestinal microbial composition among the three groups (female; male; juvenile) were analyzed by linear discriminant analysis coupled with effect size (LEfSe) analysis (LDA score > 4) and Kruskal-Wallis H test [31]. Based on the evolutionary genealogy of genes from Non-supervised Orthologous Groups (EggNOG) database and the Kyoto Encyclopedia of Genes and Genomes (KEGG) database, PICRUSt software was used to predict microbial functions and functional pathways respectively [32]. LEfSe analysis (LDA score > 2) was used to identify significantly enriched functions and KEGG pathways among the three groups (female; male; juvenile). To identify statistically significant differences of alpha diversity between the groups, Wilcoxon rank-sum test was used by SPSS 19.0 software (IBM, Armonk, USA). *P*-value < 0.05 was considered statistically significant.

## Results

### Sequencing depth and alpha diversity indices

DNA extracted from sixteen *P. canaliculata* samples were amplified successfully, and 559,712 valid sequences were obtained. *P. canaliculata* yielded 2,098 valid OTUs at a 97 % identity. By comparing the OTUs with SILVA 132 database for species annotation, 2098 OTUs were annotated to 30 bacterial phyla, 69 classes, 197 orders, 352 families, 715 genera and 1173 species. Noranked OTUs on genus level were showed in Supplementary file 1 (Datafile S1). The rarefaction curve of the Shannon index on OTU level reached asymptote (Supplementary file 3: Fig. S1), which indicated that the sequencing depth was sufficient to represent the majority of species richness in each sample.

**Fig. 1:**
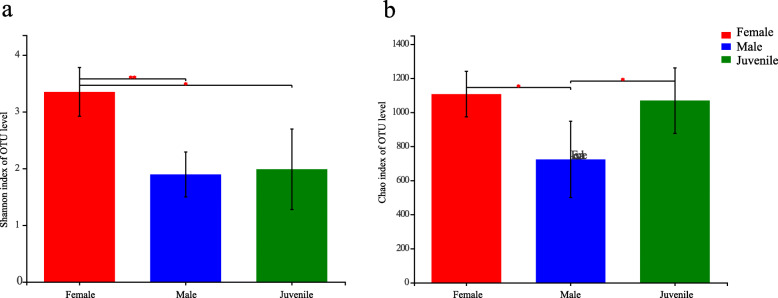
Alpha diversity index difference analysis of the female, male and juvenile group: **a** Shannon index; **b** Chao index. Significant differences were marked as “*” (0.01 < *P* < 0.05) and “**” (*P* < 0.01)

The alpha diversity measures (Shannon index and Chao diversity index) were calculated in each group to examine whether the female, male and juvenile *P. canaliculata* had differences in alpha diversity. The female group had the highest Shannon and Chao richness values, whereas the male group had the lowest bacterial richness and diversity. With reference to Shannon indices, the female group (3.35 ± SD 0.43) was significantly higher than the juvenile group (1.99 ± 0.71) (*P* < 0.05) and the male group (1.89 ± 0.40) (*P* < 0.01), while the Shannon indices of the juvenile group was not significantly different from the male group (*P* > 0.05) (Fig. 1a). With reference to Chao indices, both female group (1106.90 ± 133.68) and juvenile group (1068.7 ± 192.51) were significantly higher than the male group (723.44 ± 224.25) (*P* < 0.05). While the Chao indices between female group and juvenile group had no significant difference (*P* > 0.05) (Fig. 1b).

### Taxonomic composition and beta diversity analysis

**Fig. 2:**
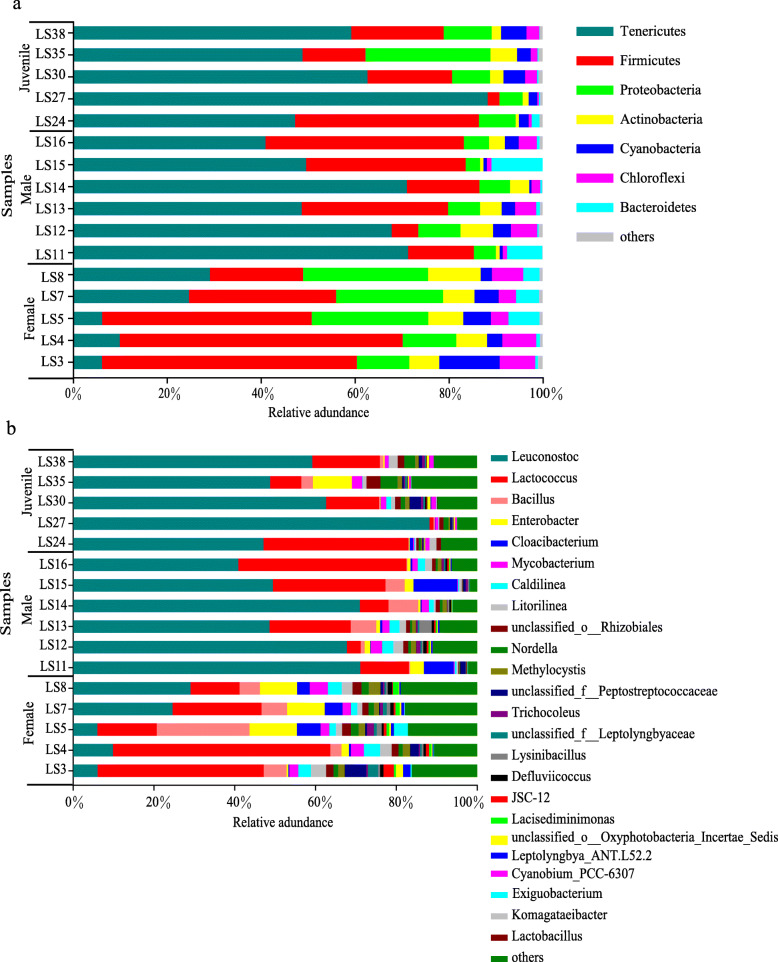
Composition of the bacterial community in the guts of female, male and juvenile *Pomacea canaliculata* snails, **a** at the phylum level; **b** at the genus level. Note: LS3, LS4, LS5, LS7, LS8 represent female *Pomacea canaliculata*; LS11, LS12, LS13, LS14, LS15, LS16 represent male *Pomacea canaliculata*; LS24, LS27, LS30, LS35, LS38 represent juvenile *Pomacea canaliculata*

In terms of prominent bacteria (relative abundance > 1 % based on classifications in any group), *P. canaliculata* harbored bacteria from seven phyla, including Tenericutes, with an average relative abundance of 45.7 %, Firmicutes (27.85 %), Proteobacteria (11.86 %), Actinobacteria (4.45 %), Cyanobacteria (3.61 %), Chloroflexi (3.4 %), Bacteroidetes (2.25 %) (Fig. 2a) and eleven genera, including *Leuconostoc* (45.68 %), *Lactococcus* (20.64 %), *Bacillus* (4.19 %), *Enterobacter* (3.25 %), *Cloacibacterium* (2.18 %), *Mycobacterium* (1.77 %), *Caldilinea* (1.52 %), *Litorilinea* (1.51 %), unclassified genus from order Rhizobiales (1.37 %), *Nordella* (1.22 %), *Methylocystis* (1.1 %) (Fig. 2b).

**Fig. 3:**
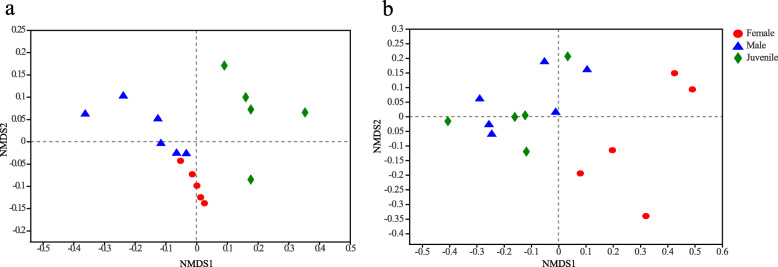
NMDS analysis based on (**a**) unweighted and (**b**) weighted Unifrac distances of gut microbiome on OTU level showing patterns of separation in gut microbiota of individuals from the female, male and juvenile group

To evaluate the overall difference in the beta diversity, NMDS analysis based on the unweighted unifrac distances showed that the three groups (female; male; juvenile) could be separated from each other. The ANOSIM analysis revealed significant differences in the structure of gut microbiota among different groups (R = 0.6282, *P* = 0.001; Fig. 3a). However, when we used weighted UniFrac distance based ANOSIM analysis to account for the abundance information, the microbiota of the male and juvenile group clustered closely and could be separated from the female group (R = 0.4131, *P* = 0.002; Fig. 3b).

AMOVA analysis was performed to further compare the intestinal microbial communities between different sex and between different developmental stages. The result showed that the intestinal microbial communities were significantly different between female and male group based on Bray-Curtis distance analyses (*P* < 0.01). The intestinal microbial communities were also significantly different between juvenile and adult group (including female and male group) based on Bray-Curtis distance analyses (*P* < 0.05).

### Microbial community similarities and differences

**Fig. 4:**
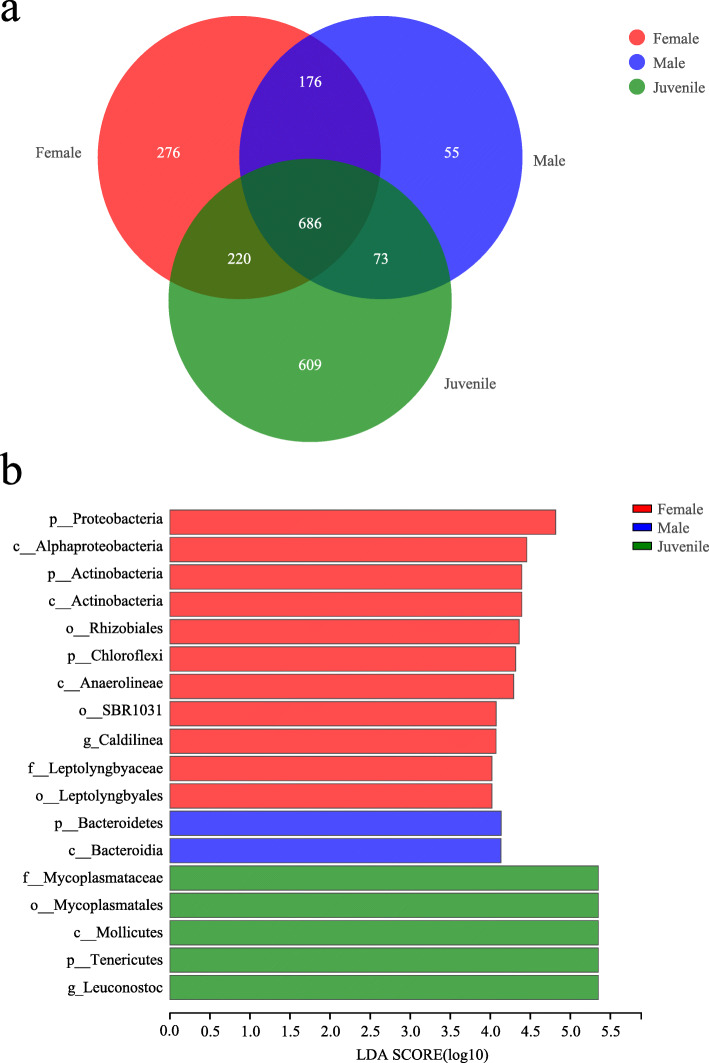
**a** Venn diagram summarizing the numbers of common and unique OTUs among the female, male and juvenile group. **b** LEfSe analysis of intestinal microbiota composition in each group (LDA > 4, *P* < 0.05). Histogram of the LDA scores computed for features differentially abundant between groups

A Venn diagram showed that a total of 696 OTUs were identified as core bacterial OTUs in all groups. The number of unique OTUs in each group was 672 (Female), 304 (Male), and 158 (Juvenile), respectively (Fig. 4a).

Female, male and juvenile *P. canaliculata* exhibited different bacterial taxa in their coiled guts (Fig. 3). According to the Kruskal-Wallis H test, we found some significant differences in the abundances of the bacterial communities among the samples from female, male and juvenile at phylum and genus levels (Supplementary file 2: Datafile S2). There were significant differences in the abundance of Tenericutes [H(3,N = 16) = 9.706, *P* = 0.008], Proteobacteria [H(3,N = 16) = 9.11, *P* = 0.011], Actinobacteria [H(3,N = 16) = 8.169, *P* = 0.017] and Bacteroidetes [H(3,N = 16) = 6.11, *P* = 0.047] in female, male and juvenile group at phylum level (*P* < 0.05). The 17 microbial genera with statistically significant differences in their abundances (*P* < 0.05) mainly included *Leuconostoc* [H(3,N = 16) = 9.706, *P* = 0.008], *Caldilinea* [H(3,N = 16) = 9.934, *P* = 0.007], *Lysinibacillus* [H(3,N = 16) = 9.993, *P* = 0.007], *Lactobacillus* [H(3,N = 16) = 11.812, *P* = 0.003]. We also performed linear discriminant analysis effect size (LEfSe) tests on the samples to detect any relative abundance differences (average relative abundance > 1 %) in the bacterial taxon (including phylum, class, order, and family). The LEfSe results were similar to the Kruskal-Wallis H test. LEfSe identified 18 discriminative features (LDA score > 4) whose relative abundance varied significantly between groups (Fig. 4b). Microbiotas of the female group were enriched in phyla Proteobacteria, Actinobacteria and Chloroflexi, classes Alphaproteobacteria, Actinobacteria and Anaerolineae, orders Rhizobiales, SBR1031, family Leptolyngbyaceae and genus *Caldilinea.* Microbiotas of the male group were enriched in phylum Bacteroidetes and class Bacteroidia. Microbiotas of the juvenile group were enriched in phylum Tenericutes, class Mollicutes, order Mycoplasmatales, family Mycoplasmataceae and genus *Leuconostoc*.

### Prediction of bacterial functions in *P. canaliculata*

**Fig. 5:**
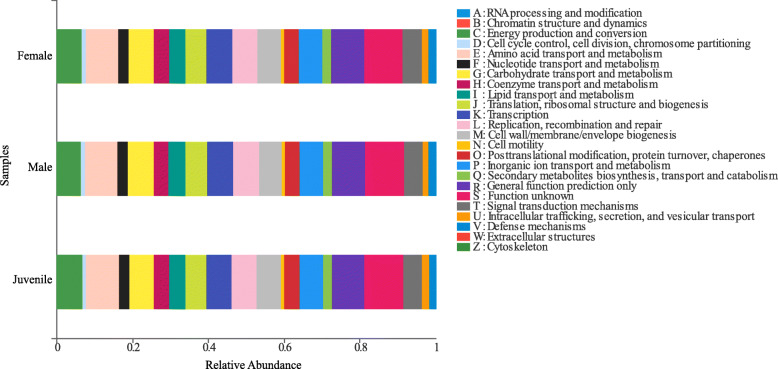
Gut microbiota predictive metabolic functions from EggNOG database in the female, male and juvenile group

The result of PICRUSt analysis based on the 16S rRNA composition data of each sample from EggNOG database showed a total of 24 metabolic functions predicted in all samples (Fig. 5). Function unknown accounted for the highest proportion (10.2 %), followed by general function prediction (8.69 %), amino acid transport and metabolism (8.6 %), transcription (6.82 %), replication, recombination and repair (6.68 %), carbohydrate transport and metabolism (6.65 %), energy production and conversion (6.53 %), cell wall/membrane/envelope biogenesis (6.19 %), inorganic ion transport and metabolism (6.15 %), translation, ribosomal structure and biogenesis (5.59 %), signal transduction mechanisms (5.04 %) and lipid transport and metabolism (4.34 %), etc. To better understand the functional differences, LEfSe test (LDA > 2) was performed in the *P. canaliculata* gut microbiotas from the three groups (female; male; juvenile). The result showed that general function prediction was enriched in the male group, while intracellular trafficking secretion and vesicular transport were enriched in the juvenile group (Supplementary file 4: Fig. S2).

In addition, predicted functional pathways were annotated using the KEGG database and a total of 5416 KEGG Orthology (KOs) were mapped to 299 level 3 KEGG pathways and were then classified into 41 level 2 KEGG pathways. Of these 41 secondary KEGG pathways, 12 pathways were involved in metabolism, 8 pathways involved in organismal systems, 6 pathways involved in human diseases, 4 pathways involved in cellular processes, 4 pathways involved in genetic information processing, 4 pathways involved in unclassified functions, and 3 pathways involved in environmental information processing. LEfSe identified 3 pathways (LDA score > 2) whose relative abundance varied significantly between groups. Environmental information processing (level 1) and secretion system (level 3) were enriched in the juvenile group, carbohydrate metabolism (level 2) was enriched in the male group (Supplementary file 5: Fig. S3).

## Discussion

The gut microbiota of animals plays an important role in food ingestion, digestion and nutrient absorption of the host [33]. During past years, only limited studies have focused on the gut microbiota of *P. canaliculata* [18, 19].

In this study, there were no dramatic differences in the bacterial composition of the female, male and juvenile group at the phylum level. The top 3 phyla in the three groups were Tenericutes, Firmicutes and Proteobacteria. The fourth abundant phylum in the intestinal contents of *P. canaliculata* was Actinobacteria by using high-throughput sequencing. Similar findings were also obtained in other snails. Proteobacteria (the relative abundance of juvenile was 36.0 %, the relative abundance of adult was 31.6 %), Firmicutes (Juvenile: 14.4 %, Adult: 6.7 %), Actinobacteria (Juvenile: 8.2 %, Adult: 12.6 %), and Tenericutes (Juvenile: 7.3 %, Adult: 6.2 %) were the predominant phyla of gut microbiota in the snail *Radix auricularia* [34]. Proteobacteria, Firmicutes, and Actinobacteria were also identified as the dominant bacterial taxa at the phylum level in the gut microbiota of adult *Oncomelania hupensis* [35].

The spatial structure of the microbiota in the intestine of female *P. canaliculata* showed that the abundance of Tenericutes was higher in the intestine than in the buccal mass and stomach [19]. Tenericutes was also the most abundant microbiota in the gut content of *P. canaliculata* in the present study. However, at the genus level, there were some differences, which might be attributed to different sample collection methods.

Firmicutes was one of the most abundant bacterial phyla in the gut content of *P. canaliculata*, which may play an important role in protein degradation [36]. *P. canaliculata* has a putative symbiont (plausibly a cyanobacterium) in the digestive gland, which travels from the stomach to the coiled gut [37–39]. The cyanobacterium produces a protease of 30 kDa that participates in the digestion of proteins [27]. In addition, Firmicutes has been reported to be able to promote preservation of gut homeostasis and host immunity development [36]. *Pomacea* has a well-developed innate cellular system and it has organs (lung and kidney) that can act as barriers against different antigens [40, 41]. In this study, some gut bacteria of *P. canaliculata* may play important roles in immunity. For example, *Lactococcus* (phylum Firmicutes; family Streptococcaceae), one of the most abundant bacterial genera in the intestinal content of *P. canaliculata*, was identified as dominant genus in lactic acid microflora of the gut of snails *Cornu aspersum* and *Oncomelania hupensis* [35, 42]. In addition, *L. lactis* subsp. *lactis* from the *Arapaima gigas* fish displayed *in vitro* antagonism against all 10 pathogens tested, including *Citrobacter freundii*, *Pseudomonas* sp., *Citrobacter freundii*, *Enterobacter* sp., *Pseudomonas stutzeri*, *Pseudomonas* sp., *Micrococcus luteus*, *Aeromonas hydrophila* ATCC 7966, *Staphylococcus agalactiae* and *Aeromonas hydrophila* DRM CPQBA 228-08 [43]. The genus *Bacillus* (family Bacillaceae, phylum Firmicutes) was identified as the core element of the intestinal microbiota of snail *Oncomelania hupensis* [35], which was also found in this study. *Bacillus cereus* is common in soil and food, and some strains are pathogenic to humans, while others act as probiotics for animals [44]. *Bacillus cereus* var. *toyoi* has been reported to be able to promote the growth and immune status of livestock and fish [45].

Proteobacteria, which was identified as the dominant phylum in the gut bacterial community of *P. canaliculata*, was also reported in the gut bacteria of other snails, such as two different terrestrial snails, *Achatina fulica*, *Helix pomatia* [46, 47], and three freshwater snails, *Biomphalaria pfeifferi*, *Bulinus africanus* and *Helisoma duryi* [48]. In previous studies, 70 % of the isolated cellulolytic bacteria from the gut of *Holotrichia parallela* larvae were Proteobacteria [49]. The genus *Enterobacter* (family Enterobacteriaceae, phylum Proteobacteria) was found in the gut of *P. canaliculata*. *Enterobacter* has been identified as cellulolytic species and reported as predominant in *A. fulica* and *H. pomatia* [46, 47], associated with carboxymethyl cellulase (CMCase) activity [46, 49, 50]. *Enterobacter* was isolated from the coiled gut of female *P. canaliculata*. Uricase specific activity could be determined in *Enterobacter.* The uric acid-degrading bacterium may participate in recycling of combined nitrogen in *P. canaliculata* [51]. Under a long period of hypometabolism, *P. canaliculata* using uric acid as a POS (Preparation for Oxidative Stress) like strategy to tolerate prolonged drought and endure low temperatures [52].

*P. canaliculata* is a voracious herbivorous snail with great environmental and ecological importance [6]. Its capacity to process a broad variety of vegetable organic matter is due to the presence of cellulolytic enzymes, both from the gut microbiome and stomach of the snail [53, 54]. The bacterial communities inside the gut of the snail may have crucial importance in the digestion of cellulose and other plant wall components [55]. Higher proportions of Proteobacteria and Firmicutes were often associated with diets containing plant ingredients [56]. Based on a culture-dependent cellulose-degrading bacteria screening method, Actinobacteria representatives could be easily recovered from the intestinal tract of *A. fulica* and could be cultivated to produce a wide range of glycoside hydrolases [11]. In addition, cellulases A0A2T7PPN6 (GHF9), A0A2T7NYY1(GHF10), A0A2T7NZR0 (GHF10), and Pc89752 (GHF10) were uniquely found in the digestive contents indicating a possible origin in unicellular glands of the gut or in commensal symbiotic organisms [57]. A bacterial cellulase secreted by *Bacillus sp*. from *Ampullaria crossean* has been characterized at the molecular and biochemical level [57, 58]. Detection of these and other cellulase-producing bacteria in the present study is consistent with the voracious herbivorous diet of *P. canaliculata.* These bacteria could help *P. canaliculata* to digest and absorb nutrients from the plant materials in their diets via the microbe production of various digestive enzymes to decompose various cellulose and hemicellulose.

There were distinct differences in the richness and diversity of the microbial community of *P. canaliculata* from the female, male and juvenile groups. Male group showed the lowest richness and diversity values and the female group showed the highest richness and diversity values.

The gut microbial analysis revealed significant differences related to developmental stages and gender indicated by NMDS analysis based on unweighted UniFrac distance and the AMOVA analysis (*P* < 0.05), indicating that sex and developmental stage have effect on gut bacterial community. In previous studies, higher ingestion rates of juvenile snails in relation to those adults have been reported in *P. canaliculata* [59]. *P. canaliculata* of small size have higher foraging and competitive abilities than big size [60]. There were some differences related to diet between juvenile and adult *P. canaliculata*. Adult *P. canaliculata* consume *Eichhornia crassipes* at a high rate while juvenile *P. canaliculata* snails do not consume this macrophyte at all [61, 62]. In addition, snails of different sizes could also use different strategies when fed on the same macrophyte species [60]. Bacterial microbiota from juvenile and adult snails were significantly different in precious reports in which the changes of the microbiota were observed at different developmental stages of the aquatic animals, such as southern catfish (*Silurus meridionalis*) and white cachama (*Piaractus brachypomus*) [63, 64], proposing that as the aquatic animal transits through youthhood to adulthood, the microbiota drifts, even when the environment remained constant.

In addition, higher growth rates, ingestion rates and growth efficiencies of female snails in relation to those of male counterparts have been reported in *P. canaliculata*, probably resulting from a higher waste of undigested food related to smaller mid-gut gland in male, which in turn results in lower food assimilation [65]. Females of *P. canaliculata* reach larger sizes than males, both in natural habitats and reared in the laboratory, a pattern that results from higher growth rates rather than from different survivorship rates [66, 67]. An indoor incubation test found that at both hungry and full states, the females had a significantly higher foraging rate than the males [68]. The higher abundance of Proteobacteria and Actinobacteria in the female group was found by LEfSe analysis. Previous studies have shown that Proteobacteria (67.13 %) and Actinobacteria (23.15 %) are representative of cellulolytic bacterial community in the hindgut of *Holotrichia parallela* larvae [49]. The phyla Proteobacteria and Actinobacteria play important roles in digesting plant and providing energy and nutrients for the host [11, 49], which may be related to higher growth rates of the female.

However, NMDS analysis based on weighted UniFrac distance showed that juvenile and male groups cluster together. This is at least in part due to abundance information, which can obscure significant patterns of variation in the taxa that are present [69, 70], indicating that taking the abundance of bacterial taxon into account reveals similarities between juvenile and male populations.

*Leuconostoc* (family Streptocnccaceae) was the most abundant genus in the gut of *P. canaliculata* and it was enriched in the gut microbiotas of the juvenile group by LEfSe analysis. *Leuconostoc* is present in many plant materials such as vegetables, silage and fermented food products and is highly beneficial to the host by fermenting various dairy products [71], which is related to the diet of *P. canaliculata* [6]. *Leuconostoc* bacteria are gram-positive, which may play important roles in the production of polysaccharides, mannitol, vitamins-K, bacteriocins and the hydrolysis of α-galactosides, which may be related to higher ingestion rates for juvenile snails than adult ones [71–74]. Recent studies have been reported that *Leuconostoc pseudomesenteroides* could significantly restore intestinal disorder caused by a high-fat diet [75].

The family Mycoplasmataceae, was also found to be enriched in the gut microbiotas of the juvenile group, related to the changes of the host environment. For example, the gut microbiome of marine *S. salar* with a higher abundance of Mycoplasmataceae was less rich and diverse than that of freshwater juveniles [13]. Moreover, KEGG pathways of environmental information processing (level 1) and secretion system (level 3), function prediction of intracellular trafficking secretion and vesicular transport were enriched in the juvenile, which might be related to the differences between juvenile and adult and could be very important in allowing juvenile to adapt to a complex digestive environment.

In the present study, Bacteroidetes was found to be enriched in the gut microbiotas of the male group. Bacteroidetes is known for fermentative metabolism and degradation of oligosaccharides derived from plant material [76]. Thus, the presence of Bacteroidetes might make an effect on the male group and allowed them to maximize the energy. Moreover, KEGG pathways of carbohydrate metabolism (level 2) were enriched in the male group. However, further study is needed to investigate the underlying mechanism on the increased Bacteroidetes in the male group. The results in our study may serve as a preliminary indication for the function of bacteria communities. Further analyses of metagenomic and metatranscriptomic approaches are required to illustrate the interactions of hosts and microbiota, as well as microbial structure and function in *P. canaliculata.*

## Conclusions

Our study provided a general understanding of the diversity characteristics of the intestinal microbial communities of *P. canaliculata*. It indicated that developmental stage and gender could both influence the intestinal microbes of *P. canaliculata.* We also found some gut microbiome associated with diets containing plant ingredients. This study can provide insight into their adaptive strategies in the environments. Further study will focus on the interaction between the gut microbiota and the host.

## Supplementary Information

**Additional file 1: Datafile S1.** Noranked OTUs on genus level using the BLASTn on NCBI nucleotide collection.

**Additional file 2: Datafile S2.** Abundance differences in the gut microbiota communities by Kruskal-Wallis H test, among the samples from female, male and juvenile at phylum and genus levels.

**Additional file 3: Figure S1.** The rarefaction curve of Shannon index on OTU level.

**Additional file 4: Figure S2.** LEfSe analysis of gut microbiota predictive COG functions in each group (LDA score>2).

**Additional file 5: Figure S3.** LEfSe analysis of gut microbiota predictive KEGG functional pathways in each group (LDA score>2)

## Data Availability

The raw data are available from the SRA database (PRJNA673528).

## Abbreviations

OTU: Operational taxonomic unit
NMDS: Nonmetric multidimensional scaling analysis
ANOSIM: Analysis of similarity
AMOVA: Analysis of molecular variance
LEfSe: Linear discriminant analysis effect size
EggNOG: Non-supervised Orthologous Groups
KEGG: Encyclopedia of Genes and Genomes
SD: Standard deviation
