# Supplementary figures and images for: Composition and diversity of gut microbiota in Pomacea canaliculata in sexes and between developmental stages
Source: BMC Microbiol. 2021 Jul 2;21:200. doi: 10.1186/s12866-021-02259-2 (PMC8252327; doi:10.1186/s12866-021-02259-2)

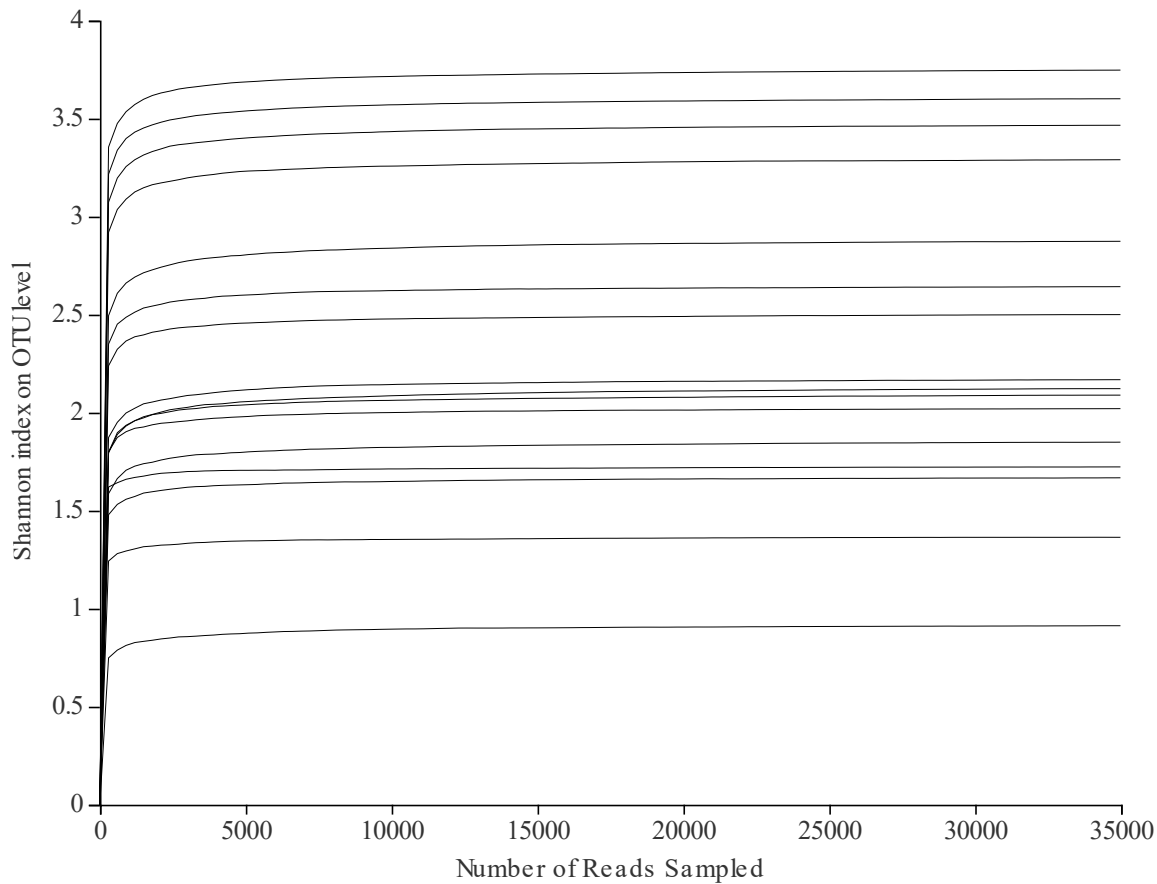

Supplement: Supplementary file 3 — Additional file 3: Figure S1. The rarefaction curve of Shannon index on OTU level. [file 12866_2021_2259_MOESM3_ESM.pdf]

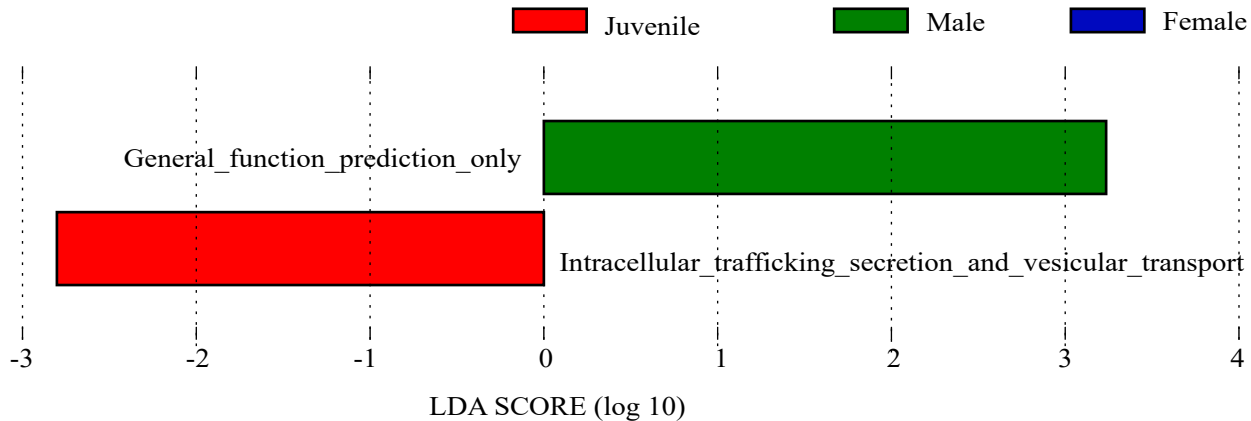

Supplement: Supplementary file 4 — Additional file 4: Figure S2. LEfSe analysis of gut microbiota predictive COG functions in each group (LDA score>2). [file 12866_2021_2259_MOESM4_ESM.pdf]

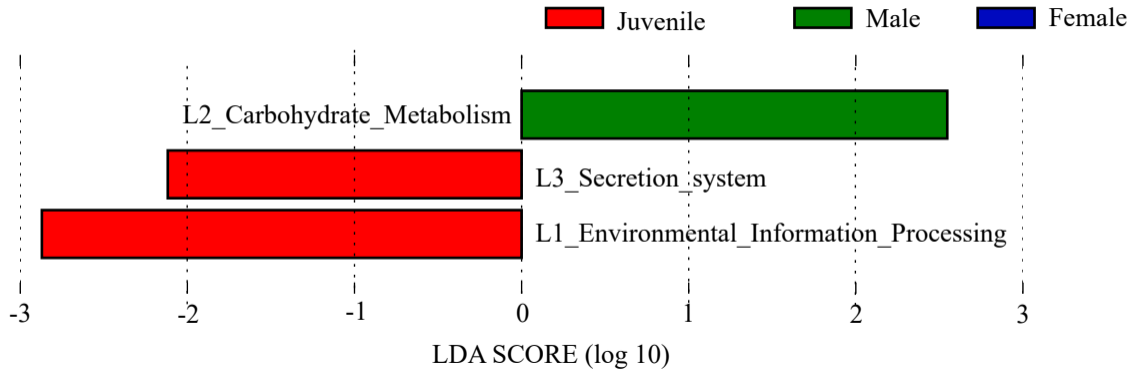

Supplement: Supplementary file 5 — Additional file 5: Figure S3. LEfSe analysis of gut microbiota predictive KEGG functional pathways in each group (LDA score>2) [file 12866_2021_2259_MOESM5_ESM.pdf]
